# Supplementary material for: Long-Interval Intracortical Inhibition and the Cortical Silent Period in Youth
Source: Biomedicines. 2023 Jan 30;11(2):409. doi: 10.3390/biomedicines11020409 (PMC9953741; doi:10.3390/biomedicines11020409)
Supplement: Supplementary file 1 [file biomedicines-11-00409-s001.zip › biomedicines-2162086-supplementary.pdf]

Supplementary Materials:

"Long-Interval Intracortical Inhibition and the Cortical Silent Period in Youth"

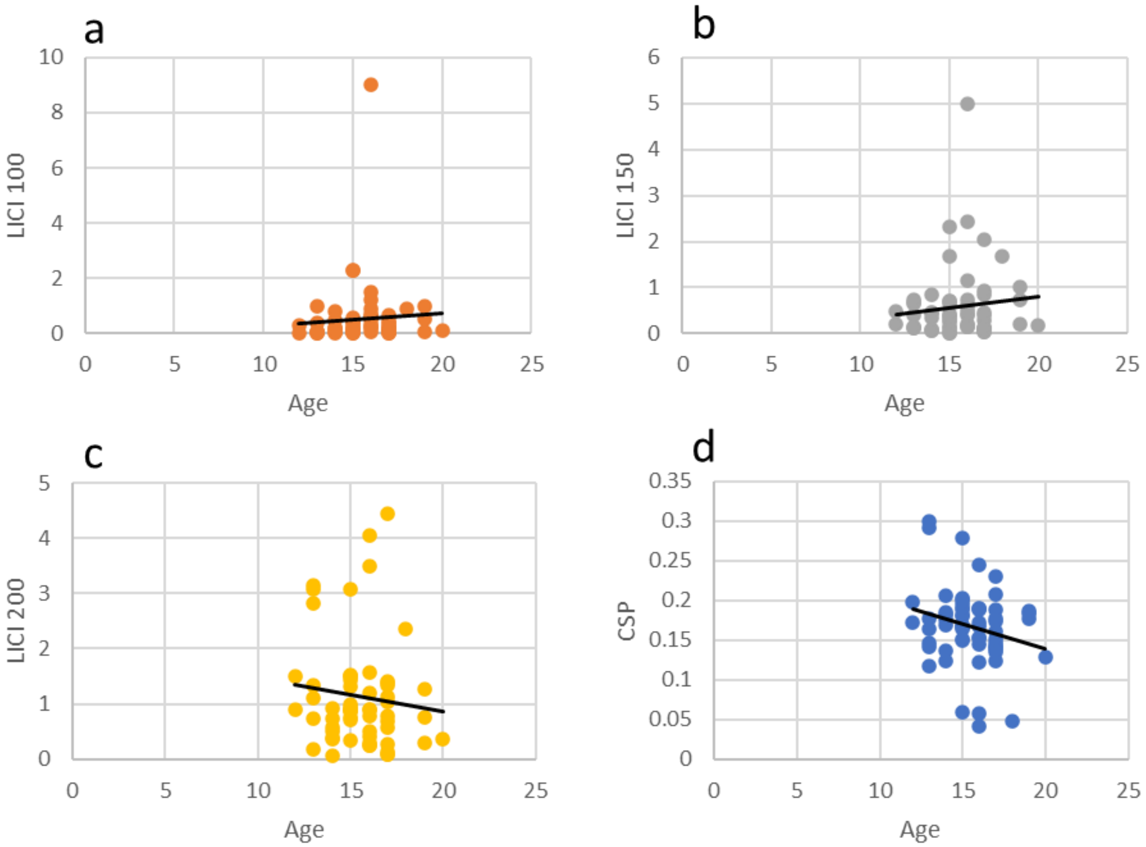

Figure S1 Relationship between age with Long-Interval Intracortical Inhibition (LICI) and Cortical Silent Period (CSP). Scatter plots of age with LICI **a)** 100 ms ISI; **b)** 150 ms ISI; **c)** 200 ms ISI and **d)** CSP paradigms.

Table S1 Association between age with cortical silent period and long-interval intracortical inhibition in youth

	Total Sample (*n*= 61)	
	**Spearman p**	***p* Value**
**LICI**		
100 ms	0.1992	0.12
150 ms	0.0763	0.55
200 ms	-0.1347	0.3
**CSP**	-0.1794	0.16
